# Metal Stent Insertion for Malignant Obstruction of a Colostomy

**DOI:** 10.7759/cureus.10121

**Published:** 2020-08-29

**Authors:** Antonios Wehbeh, Mahmoud Rahal, Hala Fatima

**Affiliations:** 1 Gastroenterology and Hepatology, Indiana University School of Medicine, Indianapolis, USA; 2 Internal Medicine, Indiana University School of Medicine, Indianapolis, USA

**Keywords:** colostomy, colonic obstruction, metal stent, colonoscopy

## Abstract

A 47-year-old female with metastatic cervical cancer and diverting colostomy presented with abdominal distention and minimal stool output from her colostomy. A computed tomography (CT) scan revealed a metastatic mass causing partial obstruction at the colostomy level and significant proximal colonic dilation. Her obstruction was relieved by the endoscopic placement of a metal stent through the stoma, with the stent’s distal edge visible externally but not protruding beyond skin level. Two months later, the stent remained patent and did not migrate. This case highlights a viable palliative treatment option for patients who are not operative candidates.

## Introduction

Malignant obstruction of colostomy is a rare occurrence that is traditionally treated with surgical intervention. However, when surgery is not a feasible option, the recommendations for its management are unclear [1]. Self-expandable metallic stents (SEMS) have been used for the relief of malignant colonic obstruction [2], yet only a few reports describing the use of stents for colostomy obstruction are available [1, 3, 4]. In this article, we report a case where a colonic stent was successfully placed to relieve malignant stomal obstruction due to metastatic cervical cancer. 

This case report was presented as a poster at the 2019 American College of Gastroenterology (ACG) Annual Meeting, San Antonio, Texas, and it was published as an accepted meeting presentation in The American Journal of Gastroenterology, October 2019, supplement issue.

## Case presentation

A 47-year-old female with a past history of hypertension and metastatic cervical cancer presented with abdominal distention and minimal stool output from her ostomy for two weeks duration. She described having small amounts of liquid stools which gradually decreased to the point of only mucous discharge from her ostomy. She had anorexia, abdominal cramping, and bloating with food intake, but no nausea or vomiting. Three years prior, she was diagnosed with stage IIB cervical cancer after presenting with abnormal vaginal bleeding. Her disease progressed rapidly despite chemotherapy and radiation. It was complicated by rectovaginal fistula for which she underwent laparoscopic diverting colostomy years prior to presentation. The patient had recently noted an enlarging mass near the ostomy site which was biopsied and showed metastatic adenocarcinoma of endocervical origin.

**Figure 1:**
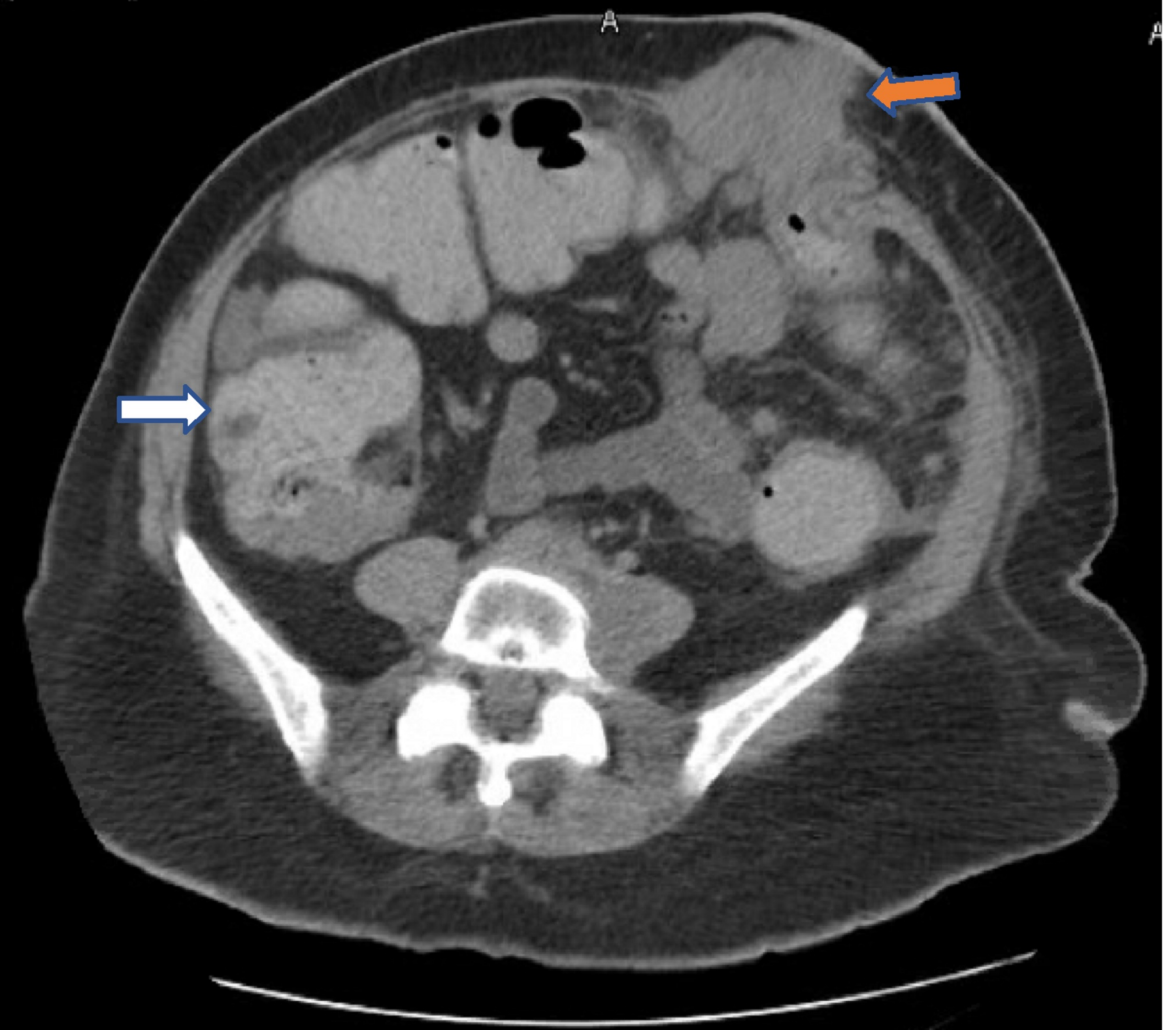
Axial cuts of an abdominal CT scan showing obstruction at the level of the colostomy by a mass (orange arrow), resulting in significant proximal colonic dilation (white arrow)

On presentation, examination revealed a soft but distended abdomen with palpable peristomal subcutaneous masses. A CT scan of the abdomen and pelvis showed a large heterogeneous mass adjacent to the colostomy with both intra-abdominal and extra-abdominal components. The mass was causing compression and partial obstruction at the level of the colostomy with significant proximal colonic dilation measuring up to 8 cm in the cecum and ascending colon (Figure 1).

As a temporizing measure, a red rubber catheter was passed through the stoma to irrigate and decompress the colon. Given the extensive metastatic disease, she was not a candidate for any operative intervention, and subsequently, endoscopic stent placement was pursued.

**Figure 2:**
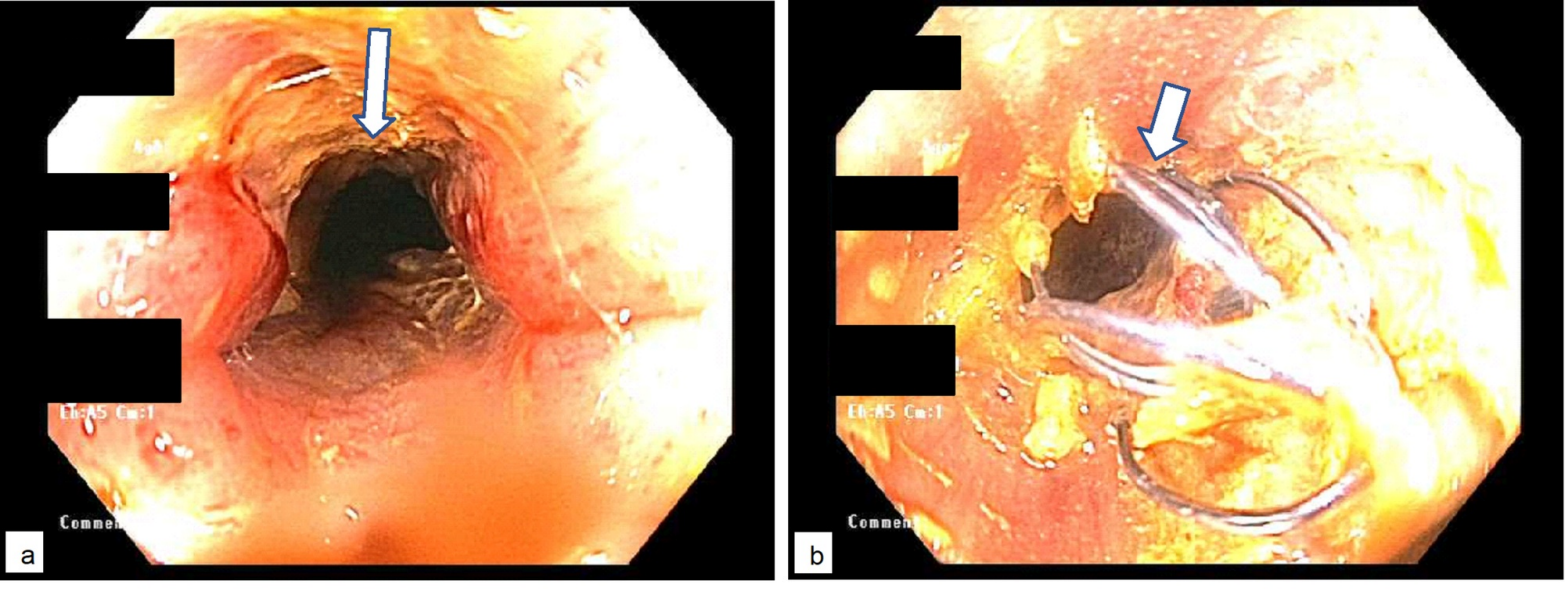
Colonoscopy showing severe stenosis at the stoma (a), treated with placement of a covered metal stent (b)

Colonoscopy was performed under moderate sedation. Examination showed a severe stenosis 4 cm in length at the surgical stoma which was traversed with the adult colonoscope. A 25 mm x 6 cm covered self-expandable metal stent (SEMS) was successfully placed with the distal edge visible externally but not protruding beyond skin level (Figures 2a, 2b).

No complications occurred during or after the procedure. After the placement of the stent, her abdominal discomfort resolved. She started to have good stool output and tolerated a regular diet.

**Figure 3:**
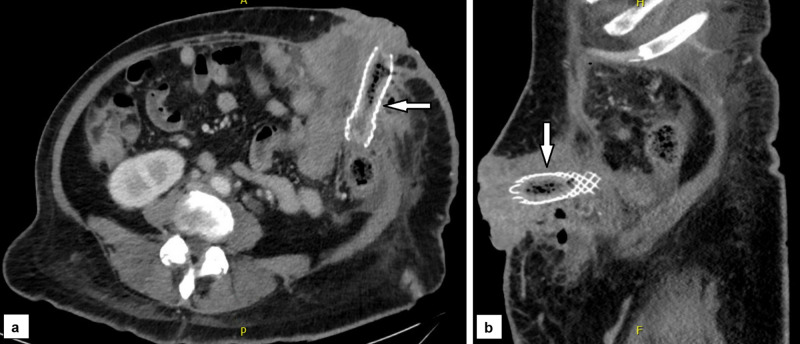
Abdominal CT scan showing the metal stent in place, as seen on axial (a) and sagittal cuts (b)

She presented two months later with draining fistula tracts around the ostomy, and a CT scan showed a patent stent still in place without recurrent colonic obstruction (Figures 3a, 3b).

## Discussion

Stoma construction is a common procedure performed in the setting of colon cancer, trauma, diverticulitis, ischemic colitis, inflammatory bowel disease, and fistulas [5]. Stenosis and malignant colostomy obstruction are among the complications of stoma construction [6, 7]. Surgery is usually the traditional treatment of these complications, however, there is limited data available regarding the management of patients who are poor surgical candidates.

SEMS can be used both as a bridge to surgery or as a palliative measure in patients with a malignant colonic obstruction due to metastatic disease and unresectable colon cancer tumor. There is no data on whether SEMS has lower morbidity or mortality when compared to palliative surgery in this group of patients. There are a few case reports describing the use of either covered or uncovered SEMS for colostomy obstruction, all of which remained patent until the patients’ death 1.5 - 6 months later [1, 3, 4].

## Conclusions

In conclusion, metal stent insertion for malignant obstruction of colostomy could be a viable palliative treatment option in patients who are not operative candidates. Further studies are needed to assess the effectiveness and safety of metal stents in improving obstructive symptoms compared with palliative surgery.
